# Genetic Profiling of Synchronous Pituitary Corticotroph Adenomas

**DOI:** 10.21203/rs.3.rs-6356485/v1

**Published:** 2025-05-05

**Authors:** Dongyun Zhang, Karen Tsai, Cristian Santana, Keanu Javaherian, Matthew Lee, Marvin Bergsneider, Kim Won, Marilene B. Wang, Harry V. Vinters, Weihong Yan, Anthony P. Heaney

**Affiliations:** University of California

**Keywords:** Cushing disease, whole exosome sequencing, single cell RNAseq, CYP21A2, GPR162, USP8

## Abstract

**Purpose:**

Double or multiple pituitary adenomas account for only 1.6–3.3% of all corticotroph tumors. We sought to better understand the underlying molecular pathogenesis of 2 distinct corticotroph adenomas that were encountered in a 43-year-old female.

**Methods:**

Two distinct histopathologically confirmed corticotroph adenomas were submitted for whole exome sequencing (WES) together with blood sample. The functional effects of identified pathogenic variants on murine corticotroph tumor pro-opio-melanocortin (*POMC*) transcription and proliferation were characterized.

**Results:**

WES demonstrated a loss-of-function variant in the G-protein coupled receptor 162 [*GPR162* (R218*)] in the right corticotroph tumor, and a novel missense variant in ubiquitin specific peptidase 8 [*USP8* (P681Q)] in the left corticotroph tumor. Compared to wild-type *GPR162* which potently suppressed POMC transcription, the premature stop-gain *GPR162* variant (R218*) found in our patient exhibited a reduced POMC transcription inhibitory effect. The novel *USP8* variant (P681Q) found in the contra-lateral tumor led to increased *POMC* transcription similar to the well characterized USP8 hotspot variant S718P. Interestingly, the patient also had a germline variant in the 21-alpha-hydroxylase gene (*CYP21A2* p.A392T) although she did not exhibit a phenotype consistent with congenital adrenal hyperplasia. The *CYP21A2* transcript and protein were absent in both corticotroph tumors from the index case whereas the protein expression was demonstrated in a series of 9 corticotroph adenomas.

**Conclusion:**

We hypothesize that the germline *CYP21A2* variant by increasing corticotroph cell stimulation may have acted in a permissive way to facilitate the additional somatic mutations which led to development of the 2 distinct corticotroph tumors.

## Introduction

Cushing’s disease is caused by excess adrenocorticotropic hormone (ACTH) secretion from a pituitary corticotroph adenoma which leads to hypercortisolism and carries a 4-fold increased mortality if untreated. The finding of occasional germline mutations in *MEN1, DICER1* and *PRKAR1A* and somatic *USP8* and rarely *NR3C1* mutations have improved our understanding of pituitary corticotroph tumorigenesis (1). The vast majority of corticotroph adenomas are single tumors. Double or multiple pituitary adenomas are very rare (2, 3, 4), occurring in only 1.6–3.3% of patients and have most frequently been observed in young to middle-aged women (4, 5, 6, 7). The molecular mechanisms of these rare multiple pituitary adenomas are poorly understood.

We encountered a 43-year-old female who presented with recent hypertension, fatigue, depression, easy bruising and a 50lb weight gain. She had had regular menses throughout her life with 3 prior uncomplicated pregnancies, no history of virilization and no family history of pituitary tumors. On exam, she had central obesity (BMI 37 kg/m^2^), a rounded plethoric face, prominent dorsocervical and supraclavicular fat pads with wide violaceous abdominal striae and diffuse bruising on her upper extremities. Her biochemical workup (summarized in Table 1) demonstrated elevated serial late-night salivary cortisol, increased 8am serum cortisol and 24-hour urine free cortisol (UFC) and failed cortisol suppression after 1mg dexamethasone with increased plasma ACTH consistent with ACTH-dependent Cushing syndrome. Of note, she had a normal 17-hydroxy progesterone level.

**Figure 1:**
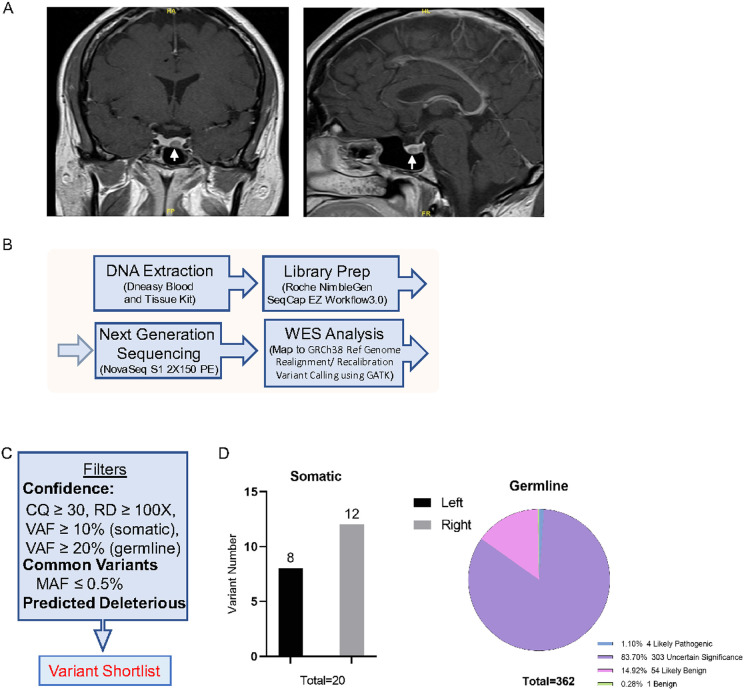
MRI images of index case and introduction of sequencing and analysis workflow. (A) Contrast-enhanced T1-weighted coronal (right) and sagittal (left) MRI images in the index case demonstrating left-sided hypoenhancing lesion (arrowed). (B) Schematic workflow summarizing the steps in pituitary tumor retrieval and whole-exome sequencing and data processing using automated pipelines in the index case corticotroph tumors. (C) Illustration of the analytic Alters used to identify somatic and germline variants. (D) Summary of identified somatic and germline variants.

Although pre-operative MRI of the pituitary gland showed a single 8x6mm left sided pituitary microadenoma (Fig. 1A), at trans-nasal trans-sphenoidal surgery the 8mm left sided pituitary microadenoma was identified and removed along with a separate distinct 7mm right sided pituitary microadenoma. The surgeon documented that the two tumors were clearly separated on far sides of the pituitary gland. Her 8am serum cortisol fell to 1μg/dL the following morning in keeping with immediate remission and she therefore commenced glucocorticoid replacement with hydrocortisone 10mg at 8am and 5mg at 12-2pm. Histopathological examination confirmed 2 distinct ACTH-immunopositive pituitary tumors. Ki-67 in both was < 1% and both corticotroph tumors exhibited prominent Crooke’s hyaline change consistent with Crooke’s cell adenomas.

Given the unusual concurrence of 2 corticotroph adenomas, FFPE samples of both the left and right pituitary corticotroph tumors and matched patient blood were subjected to whole exome sequencing (WES) (summarized schematically in Fig. 1B). A total of 259 million paired-end reads of 150bp length (left tumor 88M, right tumor 101M and blood 69M, Supplementary Table-1) were obtained (8).

## Materials and Methods

### DNA Isolation and Whole Exome Sequencing

This study complies with the Declaration of Helsinki. In accordance with institutional human subject guidelines at UCLA (IRB# 20-002235), informed written consent for use of biological samples for research purposes was obtained from all subjects before surgery. Pathological evaluation was performed by a dedicated neuropathologist (H.V.), who identified, and marked areas of tumor. Thereafter trimmed FFPE specimens from both the right and left corticotroph tumors (> 85% tumor purity), along with peripheral blood were submitted for genomic DNA isolation using DNeasy Blood and Tissue kit (Qiagen, USA) (9). Genomic DNA quality (gDNA) was checked using the Agilent Test Genomic DNA Analysis, and gDNA quantity was measured using Nanodrop and Qubit fluorometer 3.0. Two hundred nanograms of genomic DNA were fragmented, end repaired, phosphorylated and ligated to barcoded sequencing adaptors for library preparation according to Roche NimbleGen SeqCap EZ Workflow 3.0. The sequencing was performed using NovaSeq S1 (2x150 PE) at UCLA Technology Center for Genomics & Bioinformatics (TCGB). Raw data are deposited in NCBI BioSample database with SAMN47584567 (left tumor), SAMN47584568 (right tumor), SAMN47584569 (blood).

### Bioinformatic Analysis of WES Data

The sequence data were aligned to the GRCh38 human reference genome using Partek_flow bwa-0.7.17 (bwa mem) (10). PCR duplicates were marked using Mark Duplicates program in picard_tools/2.13.2 tool set. GATK v4.0.8.1 was used for insertions and deletions (INDEL) realignment and base quality recalibration (11). The GATK HaplotypeCaller was applied to germline sequence BAM to identify germline single nucleotide polymorphisms (SNPs). VarScan2 (12) were used to call the single nucleotide variants (SNVs) and small INDELs between normal vs tumor pairs. All variants were annotated using the WAnnovar program (13).

### Identification of Germline and Somatic Variants

The resultant VCF files were uploaded into the Qiagen Clinical Investigation (QCI) pipeline for filtration. Germline variants were considered confident if they had a GATK genotype quality (GQ) score ≥ 50, variant allele frequency (VAF) ≥ 20%, and depth of coverage ≥ 100x (14). Common variants were excluded if population minor allele frequency (MAF) in population databases such as the 1000 Genomes Project, gnomAD, CGI, the National Heart, Lung and Blood Institue (NHLBI) Grand Opportunity (GO) Exome Sequencing Project, and Exome Aggregation Consortium (ExAC) was ≥ 0.5% (15). The functional effects of the mutations were predicated using scores of Sorting Intolerant From Tolerant (SIFT, ≤ 0.05), PolyPhen-2 (≥ 0.85), and Combined Annotation Dependent Depletion (CADD, ≥ 15) (16, 17, 18). The variants listed in HGMD^®^, ClinVar, and CentoMD^™^ were all included (19). Shortlisted variants were then validated by visualization of aligned data using integrated genomics viewer (IGV). Germline variants were validated by comparing variants identified in both pituitary tumors and blood samples. Variants specifically observed in tumor DNA but completely absent in germline DNA were considered as somatic variants. Annotation of identified somatic mutations was carried out using Qiagen QCI software with variant allele frequency (VAF) ≥ 10%, depth of coverage ≥ 100x, and minor allele frequency (MAF) ≤ 0.005 from population studies unless listed as somatic in COSMIC (20). Filtering of common variants and predicted deleterious variants followed the same criteria for germline variants. CYP21A2 RNA expression was compared using scRNAseq dataset, which were deposited in NCBI BioSample database with BioSample accessions of SAMN18316925 (CD1), SAMN18316926 (CD2), SAMN18316928 (CD3), SAMN18316929 (CD4) (21), and SAMN47620853 (CD5).

### Histopathological Analysis

To quantitate CYP21A2 IHC staining, a CYP21A2 antibody ((Sigma-Aldrich Cat# HPA048979, RRID:AB_2680584) was incubated overnight at 4 °C with 5-μm tumor sections from the index case and an in-house built pituitary tumor tissue microarray (TMA) which contained 9 corticotroph tumors. CYP21A2 immunostaining intensity was scored using the histoscore (range, 0 to 400), derived from the product of staining intensity (absent, 0; weak, 100 to 200; intermediate, 200 to 300; strong, 300 to 400) and the percentage of tumor cell staining (range, 0 to 100). All immunocytochemistry results were assessed in blinded fashion by our neuropathologist (H.V.).

### Cell Culture, SiRNA, and Plasmid Transfection

AtT20/D16V-F2 murine corticotroph tumor cells secreting ACTH were purchased from ATCC (Cat# CRL-1795, RRID:CVCL_4109) and cultured as monolayer at 37°C, 5% CO2 using Dulbecco’s Modified Eagles Medium (DMEM, Thermo Fisher Scientific, Hanover Park, IL) containing 10% fetal bovine serum (FBS, Sigma), and penicillin/streptomycin (Thermo Fisher Scientific, Hanover Park, IL). The cultures were detached with trypsin and transferred to new 75-cm2 culture flasks twice a week. On-target plus mouse GPR162 siRNA was purchased from Horizon (Cat# L-061617-00-0005), and Non-targeting Pool (Cat# D-001810-10-05) was used as siRNA control. The plasmids containing GPR162 (NM_013533) coding region (pCMV6-Entry-GPR162, Cat# MR209174) and USP8 (NM_001128610) coding region (pCMV6-Entry-USP8, Cat# RC226336), or empty vector (pCMV6-Entry, Cat# PS100001) were purchased from OriGene Technologies, Inc. (Rockville, MD). GPR162 R218* and USP8 P681Q and S718P mutations were customized by Azenta US, Inc. All sequences were confirmed by automatic DNA sequencing. Murine corticotroph tumor AtT20 cells were transfected with siRNA GPR162, pCMV6-Entry-GPR162-WT and R218*, or pCMV6-Entry-USP8-WT, P681Q and S718P vectors using Lipofectamine 2000 (Life Technologies, Inc).

#### Cell Viability Assay

The effect of GPR162 and USP8 variants on AtT20 cell viability was assessed by the CellTiterGlo assay (Promega, Cat# G7573). The transfectants as indicated above were suspended in 100 μl DMEM supplemented with 10% FBS and plated in 96-well plates (2×103 viable cells/well) and cultured overnight. Results are presented as mean ± standard error proliferation index (relative luminescence signal to medium control) compared to siRNA control, or empty vector, and all experiments were repeated at least three times.

#### Real-Time PCR

Murine corticotroph tumor AtT20 cells were transfected with pCMV6-Entry-USP8-WT, P681Q and S718P expression constructs, or pCMV6-Entry-GPR162-WT and R218* constructs. Following treatment with Dex (10 and 100nM) for 24hrs, the total RNA was extracted with RNeasy kit (Qiagen). RNA quantification and integrity were assessed by measurement of absorbance at 260 and 280 nm. Total RNA was reverse transcribed into first-strand cDNA using a cDNA synthesis kit (Invitrogen). Quantitative PCR reactions were carried out using CFX Real-time PCR Detection System (Bio-Rad Laboratories Inc.). Primer sequences (Invitrogen/Life Technologies) were as follows: mouse β-actin forward primer, 5'-GGC TGT ATT CCC CTC CAT CG-3'; mouse β-actin reverse primer, 5'-CCA GTT GGT AAC AAT GCC ATG T-3'; mouse POMC forward primer, 5'- CCA TAG ATG TGT GGA GCT GGT G-3'; mouse POMC reverse primer,5'-CAT CTC CGT TGC CAG GAA ACA C-3'; mouse GPR162 forward primer, 5’-AGC TTC TTC TCC TTG AAG TCA GAC-3’; mouse GPR162 reverse primer, 5’-TCA CAG GTG CCA TCG TCA TCA-3’.

#### Immunoprecipitation and Western Blotting.

AtT20 cells were transiently transfected with GFP-EGFR (gifted by Alexander Sorkin (Addgene plasmid# 32751 ; http://n2t.net/addgene:32751 ; RRID:Addgene_32751) (22) in combination with USP8-WT, P681Q and S718P mutations as well as empty vector (pCMV6-Entry) at 1:1 ratio. 24h after transfection, cells were treated with EGF (100ng/mL) for 5 min, then lyzed in Cell Lysis Buffer (Cell Signaling, Cat# 9803), containing 20 mM Tris-HCl (pH 7.5), 150 mM NaCl, 1 mM Na_2_EDTA, 1 mM EGTA, 1% Triton, 2.5 mM sodium pyrophosphate, 1 mM beta-glycerophosphate, 1 mM Na_3_VO_4_, 1 μg/ml leupeptin. Cell lysates were collected after centrifugation at 4C for 30 min, and precleared using 50uL of Protein A/G PLUS-Agarose beads (Santa Cruz, Cat# sc-2003) at 4C for 3hrs. Protein concentration of precleared cell lysates was determined by DC Protein Assay Kit II (BioRad Cat# 5000112EDU). 500ugs of cell lysates was incubated with anti-GFP antibody linked agarose beads (30uL per sample, Vector Laboratories, Cat# MB-0372) for immunoprecipitation at 4C overnight. The precipitated complex was washed three times with cell lysis buffer and eluted with 2x sample buffer (Fisher Scientific, Cat# AAJ61337AC). Elutes were analyzed by Western Blotting using 8% Bis-Tris PAGE gel (GeneScript, Cat# NC1438344) and MOPS buffer (Fisher Scientific, Cat# NC1278898). Primary antibodies for immunoblotting included anti-GFP (Cell Signaling Technology Cat# 2956, RRID:AB_1196615), anti-Ubiquitin (Cell Signaling Technology Cat# 20326, RRID:AB_3064918), anti-Myc-tag (Cell Signaling Technology Cat# 2276, RRID:AB_331783), and anti-Actin (Santa Cruz Biotechnology Cat# sc-47778, RRID:AB_626632). Secondary peroxidase-conjugated anti-mouse IgG (Cell Signaling Technology Cat# 7076, RRID:AB_330924) and anti-rabbit IgG antibodies (Cell Signaling Technology Cat# 7074, RRID:AB_2099233) were used. Blots were detected using SuperSignal^™^ West Femto Maximum Sensitivity ECL Detection Reagents (Thermo Scientific, Cat# PI34095), and visualized using iBright imaging system (Thermo Scientific, Cat# CL750). The blots shown were representative of three experiments.

#### Statistical Analysis

PCR results and intensity of CYP21A2 staining in the corticotroph pituitary tumors weres compared using the Mann-Whitney U-test by GraphPad Prism 8 (GraphPad Software) taking p values < 0.05 as significant.

## Results

### Somatic Variants

Somatic variants were identified in the corticotroph tumors by comparing tumor variants with the patient’s constitutional genomic DNA. After filtering for variant calling confidence with a call quality (CQ) score ≥ 30, read depth of coverage (RD) ≥ 100x (14), variant allele frequency (VAF) ≥ 10%, minor allele frequency (MAF) in normal population databases ≤ 0.5% (15), and biological deleterious variants (Fig. 1C), we identified 20 putative tumor related variants (8 in left tumor and 12 in right tumor, Fig. 1D, and Supplementary Table-1) (8). Since these variants were of uncertain significance, we used Combined Annotation Dependent Depletion (CADD) to predict their possible functional effects. The highest CADD scores were observed for a variant in the G protein coupled receptor 162 [*GPR162* (NM_019858.2, c.652A > T p.R218*)] in the right tumor (CADD score 40) and for a variant in ubiquitin specific protease 8 [*USP8* (NM_001128610.3, c.1724C > A p.P681Q)] in the left tumor (CADD score 31), respectively (Supplementary Table-2) (8).

### Right Corticotroph Tumor

**Figure 2:**
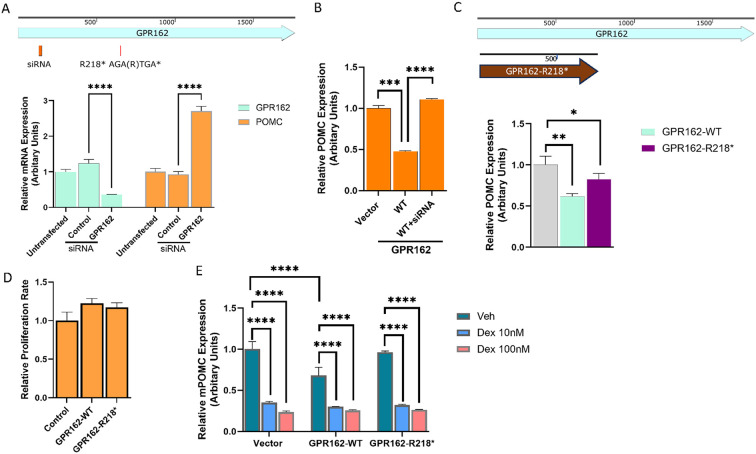
Functional studies of the somatic variant GP162 R218* identified in the right tumor. (A) Murine corticotroph tumor AtT20 cells were transiently transfected with siRNA GPR162 and siRNA control. GPR612 knockdown efficiency (left) and *POMC* mRNA expression (right) were analyzed by real time PCR. (B) AtT20 cells were transiently transfected with constructs expressing wild type (WT) GPR162 and vector controls. Twenty-four hours later, the second round of transfection was performed to introduce siRNA GPR162 for rescue experiments. *POMC* mRNA expression was analyzed by real time PCR. (C & D) Constructs expressing wild type (WT) and GPR 162 (R218*) variant were transiently transfected into AtT20 cells. The effects of the ectopic expression of WT and mutant GPR162 on basal *POMC* mRNA expression were assessed by real time PCR (C), and cell proliferation rate was assessed by CellTiterGlo assay (D). (E) Dex-induced POMC inhibition were evaluated by real time PCR following transient transfection of WT-GPR162 and R218*. The Dex treatment was at 10nM and 100nM for 24h, and DMSO was used as vehicle control.

The *GPR162* c.652A > T variant identified in the right tumor with 10% VAF (of 276 reads) was predicted to cause a stop gain at p.R218* in exon 2. *GPR162* is an orphan G protein coupled receptor of the Rhodopsin family which is highly expressed in the cerebellum, hindbrain, hypothalamus and pituitary, and is associated with food consumption and energy homeostasis (23, 24, 25, 26). To explore a role, if any, of *GPR162* in regulation of corticotroph tumor *POMC* expression and proliferation, we first knocked down *GPR162* expression in the murine corticotroph tumor AtT20 cells using a specific siRNA targeting a region (ACACAAGCCACUGGAGUUA) in Exon 1 (± 0.358, p < 0.0001, Fig. 2A). GPR162 knockdown resulted in increased *POMC* mRNA expression compared to mock-SiRNA and untransfected control (2.708 ± 0.131, p < 0.0001, Fig. 2A). In contrast, transient overexpression of wild type (WT) GPR162 resulted in reduced *POMC* mRNA expression (0.48 ± 0.008, p < 0.005, Fig. 2B). This effect of GPR162 overexpression on POMC mRNA expression was rescued by co-transfection of an siRNA to GPR162 (1.10 ± 0.01, Fig. 2B), confirming the specific effect of GRP162 on POMC regulation. Transfection of the GPR162 variant R218* that we had demonstrated in our index patient also resulted in reduced corticotroph tumor *POMC* mRNA expression (0.8 ± 0.07, p < 0.05, Fig. 2C) but this was not as marked as overexpression of the GPR162-WT (0.6 ± 0.03, p < 0.05, Fig. 2C). In contrast to its actions on POMC transcription, GPR162 overexpression did not alter corticotroph tumor cell proliferation (Fig. 2D) or actions of Dexamethasone to suppress POMC transcription. As noted before basal POMC mRNA expression was lower in corticotroph tumor GPR162-WT transfectants (0.68 ± 0.1, p < 0.05, Fig. 2E), but both the GPR162-WT and the GPR162-R218* corticotroph tumor transfectants exhibited a similar reduction in POMC mRNA following Dex treatment (10, and 100nM for 24h) (Control vs. GPR162-WT vs. GPR162-R218* Dex 10nM: 0.35 ± 0.013 vs. 0.3 ± 0.005 vs. 0.32 ± 0.01; Dex 100nM: 0.24 ± 0.015 vs. 0.26 ± 0.009 vs. 0.26 ± 0.003, Fig. 2E). This latter finding indicates that *POMC* transcriptional responsivity mediated by the glucocorticoid pathway does not appear to be affected by either GPR162-WT- or the GPR162-R218* variant.

### Left Corticotroph Tumor

**Figure 3:**
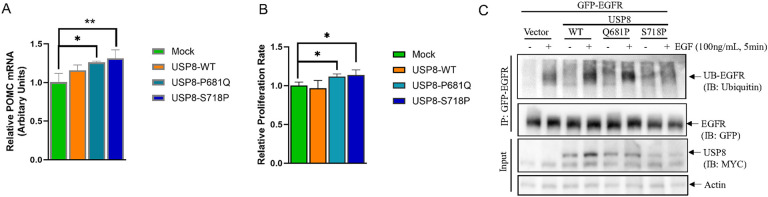
Role of USP8 variant Q681P identified in the left tumor in POMC regulation and EGFR ubiquitination. (A & B) Wild type and mutant USP8 (P681Q and S718P) plasmids were transiently transfected into AtT20 cells, and the changes in *POMC* mRNA expression (A) and proliferation rate (B) were assessed by real time PCR (A) and CellTiterGlo assay (B). (C) AtT20 cells were transiently transfected with GFP-EGFR in combination with either WT-USP8, P681Q, S718P variants or empty vector. Twenty-four hours later, corticotroph tumor cell transfectants were treated with (+) or without (−) EGF (at 100ng/mL for 5 min). EGFR ubiquitination was detected by immunoprecipitating with anti-GFP antibody linked agarose bead, and immunoblotting with the indicated antibodies.

The contra-lateral (left) corticotroph tumor from our patient harbored a novel *USP8* c.1724C > A variant which causes a missense P681Q with 18.7% VAF (of 187 reads), which was further validated by amplicon NGS sequencing (23.8% VAF, 146 out of 614 reads). *USP8* gain-of-function mutations have been identified in ~ 30–50% of corticotroph tumors with a female predominance (27, 28, 29). Overexpression of the *USP8* P681Q variant that we had found in our patient in murine corticotroph tumor cells caused an increase in *POMC* mRNA expression which was comparable to the *USP8* hotspot S718P mutation (USP8 P618Q vs. USP8 S718P fold change: 1.26 ± 0.014 vs. 1.31 ± 0.11, Fig. 3A). Furthermore, both the *USP8* P618Q mutation identified in our patient and the hotspot S718P variants increased murine corticotroph tumor cell proliferation (USP8 P618Q vs. USP8 S718P fold change: 1.12 ± 0.03 vs. 1.14 ± 0.07, p < 0.05, Fig. 3B). Given prior studies implicating USP8 in EGFR ubiquitination, we evaluated EGFR immunoprecipitation following corticotroph tumor EGF-treatment (30). GFP-tagged EGFR in combination with Myc-tagged WT-USP8, USP8-P681Q variant, and the USP8-S718P variant as well as empty vector were transiently transfected into corticotroph tumor cells followed by immunoprecipitation with anti-GFP antibody linked agarose beads. Following treatment with EGF (100ng/mL, for 5min), ubiquitination of GFP-EGFR was observed in WT-USP8 transfectants. In contrast, ubiquitinated EGFR levels were reduced in the USP8-S718P variant transfectants (Fig. 3C) whereas overexpression of the novel USP8-P681Q variant did not appear to abrogate EGFR ubiquitin. This suggests the USP8-P681Q variant regulates POMC transcription by an alternate mechanism to ubiquitination of EGFR, similar to that described for other USP8 variants (28).

### Germline Variants

**Figure 4:**
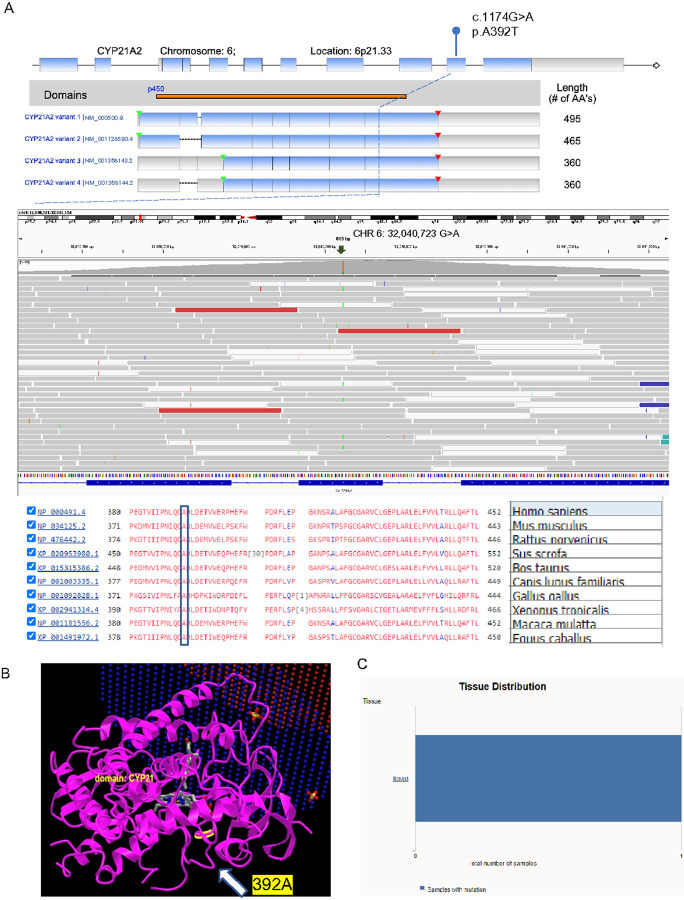
Illustration of features of germline variant of CYP21A2. (A) Location of amino acid changes caused by the CYP21A2 c.1174G>A (p. A392T) germline variant identified in the index case. (B) Depiction of the 3-dimensional structure of CYP21A2 crystal structure and highlighted site of identified CYP21A2 variant. (C) The list of this variant in COSMIC dataset.

Germline variants were considered confident if they had a call quality (CQ) score ≥ 50, read depth of coverage (RD) ≥ 100x (14), and variant allele frequency (VAF) ≥ 20%. Common variants were excluded if the minor allele frequency (MAF) in normal population databases was ≥ 0.5% (15) (Fig. 1C). Variants that were detected in the patient’s blood sample were also cross-checked in both tumor samples using VarScan2 pipeline. We detected 362 rare genetic variants, amongst which 4 (1%) were likely pathogenic, 303 (84%) were of uncertain significance, 54 (15%) were likely benign, and 1 was benign (Fig. 1D, and Supplementary Table-3) (8). Of the 4 likely pathogenic germline variants, a *CYP21A2*c.1174G > A variant was the most commonly detected with a 20.28% VAF (of 572 reads) causing a missense variant at p.A392T (Supplementary Fig. 1) (8) which was also confirmed by Sanger sequencing. *CYP21A2* encodes steroid 21-hydroxylase which plays an important role in steroid biosynthesis by converting progesterone and 17-hydroxyprogesterone to deoxycorticosterone and 11-deoxycortisol respectively (31, 32). The A392 residue is located in the 9th exon of the Cytochrome P450 domain and is present in all 4 human *CYP21A2* transcript isoforms (NM_000500.9, NM_001128590.4, NM_001368143.2 and NM_001368144.2, Fig. 4A). It is highly conserved amongst vertebrate CYP21 orthologs (Fig. 4A). The A392 residue is located at the C-terminal inner core of the protein (Fig. 4B) and predicts a change from a small nonpolar alanine residue to the bulkier polar threonine residue (A→T) which changes protein hydrophobicity and its overall structure. More than 20 germline *CYP21A2* variants cause congenital adrenal hyperplasia (CAH) (33) and the A392T missense variant was first identified in a pediatric female patient with non-classical CAH and symptoms of mild androgen excess (34). Functional studies have demonstrated that the A392T variant moderately reduces 21-hydroxylase activity to 38.7% (SD = 9.5%) of normal for 17-hydroxy progesterone and to 22.9% (SD = 4.7%) of normal for progesterone (34). This *CYP21A2* A392T variant is listed in COSMIC (COSM 6353585), and has also been previously reported in a patient with breast carcinoma (COSU669, Fig. 4C).

#### Validation of CYP21A2 Expression at RNA and Protein Levels.

**Figure 5:**
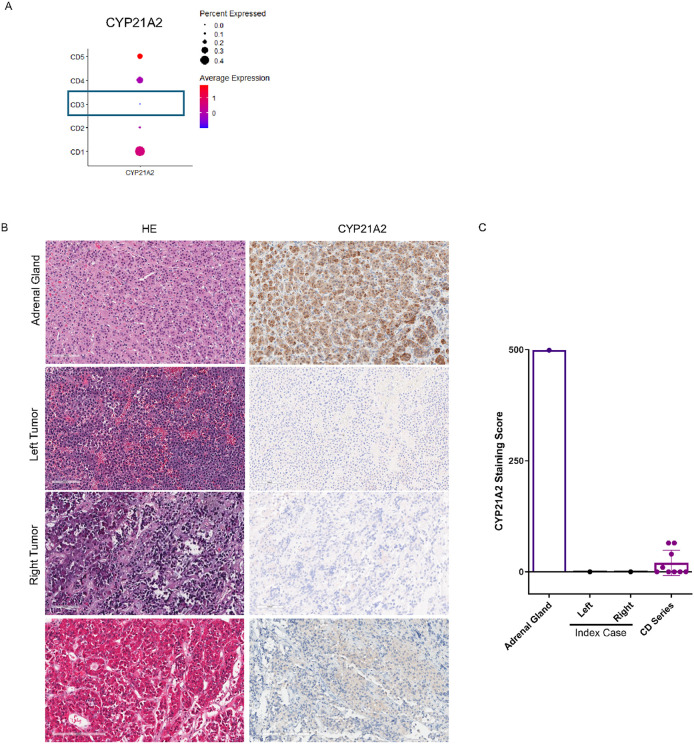
Validation of CYP21A2 expression at RNA and protein levels. (A) scRNAseq demonstrated CYP21A2 mRNA expression in 4 additional sporadic corticotroph tumors (CD1, 2, 4 & 5), but absent CYP21A2 transcript in the index corticotroph tumor (CD3). (B) Using normal adrenal gland as positive control, immunocytochemical CYP21A2 expression was found to be absent in both corticotroph tumors from the index case, but present in 9 additional sporadic corticotroph tumors. magnification x 100. (C) CYP21A2 immunostaining intensity was scored using the histoscore (range, 0 to 400), derived from the product of staining intensity (absent, 0; weak, 100 to 200; intermediate, 200 to 300; strong, 300 to 400) and the percentage of tumor cell staining (range, 0 to 100).

To determine the specificity of the *CYP21A2* germline variant, given its relationship to the hypothalamic-pituitary–adrenal axis, we compared *CYP21A2* RNA expression in our index case with 4 additional randomly selected sporadic corticotroph tumor samples (35). As shown in Fig. 5A, *CYP21A2* RNA expression was uniquely absent in the index case (CD3) compared to the other corticotroph tumors which exhibited a range of *CYP21A2* RNA expression. We confirmed this finding by immunocytochemistry for *CYP21A2* protein, using a normal human adrenal gland as a positive control (Fig. 5B, Upper Panel). As illustrated in Fig. 5B (Middle & Lower Panels) *CYP21A2* immunostaining was not detected in either the right or left pituitary corticotroph tumors derived from the index patient, supporting our WES and transcriptional studies. In contrast, *CYP21A2* immunopositivity was seen in 9 additional sporadic corticotroph tumors (Fig. 5C).

## Discussion

This is only the fourth documented case of double pituitary ACTH-secreting corticotroph adenomas. Two prior cases were similar to our own in that the corticotroph adenomas expressed only ACTH. One occurred in a 56-year-old female with Cushing’s disease who had a 2mm pituitary corticotroph microadenoma close to the neurohypophysis and a second clearly distinct 9mm right sided sellar corticotroph microadenoma (26). The other case was a 38-year-old female with Cushing’s disease who had a corticotroph adenoma located in the pituitary gland with an additional corticotroph adenoma in the pituitary stalk (28). The third case occurred in a 50-year-old female with Cushing disease who had 2 sellar lesions on pituitary MRI imaging. Both the resected tumors exhibited ACTH immunopositivity along with focal positivity for TSH, GH, and PRL (27). These cases and our own are instructive in several respects. Firstly, they emphasize the importance of careful pre-operative radiologic review and surgical exploration of the pituitary gland to locate and remove these albeit rare multiple pituitary adenomas to optimize the chance of complete disease remission. They also illustrate the limitations of current imaging approaches, where despite advances, detection of microadenomas < 3mm adenomas remains challenging (6, 36, 37). The underlying cause of multiple pituitary adenomas is unclear but three hypotheses have been offered. The so-called “multiple-hit theory” proposes coincidental expansion of 2 distinct genetically mutated pituitary cell types (6). In contrast, the “trans-differentiation theory” proposes that cells of one pituitary adenoma transdifferentiate into another cell type with different morphologic and phenotypic characteristics (38). Finally, the “induction theory” postulates that one adenoma in some way can induce the formation of another (39).

Advanced next generation sequencing (NGS) techniques such as whole exosome sequencing (WES) have identified novel mutations that participate in pituitary tumor pathogenies (42, 43, 44). In corticotroph tumors, somatic mutations in the ubiquitin-specific peptidase 8 (*USP8*) gene have been identified in 30–60% of cases (27, 28, 29). Of 17 different *USP8* somatic variants identified to date (27), a mutation hotspot has been highlighted in exon 14 (between aa713 and aa720) within a 14-3-3 binding motif (27, 28). We now describe a novel missense *USP8* variant c.1724C > A in the left tumor of our patient which when overexpressed, resulted in increased *POMC* transcription, comparable to that observed with the hotspot *USP8* mutation S718P. The hotspot mutations in the exon 14-3-3 binding region is reported to facilitate proteolytic cleavage at the site of ^714^KR^175^ and activation of USP8 (30). However, the *USP8* P681Q variant did not affect EGFR ubiquitination suggesting a different mechanism by which it regulates POMC transcription. Interestingly, we also identified a loss-of-function variant in the *GPR162* gene in our patient’s right corticotroph tumor. Functional studies in murine corticotroph tumor cells demonstrated that *GPR162* negatively regulates P *OMC* mRNA expression and the GPR162 variant R218* that we had demonstrated in our index patient also resulted in reduced corticotroph tumor *POMC* mRNA expression. Clearly, further studies are needed but our findings identify *GPR612* as a novel regulator of corticotroph tumor POMC transcription.

Of the 4 likely pathogenic germline variants identified by our WES studies, the germline variant in CYP21A2 stood out to us as it is intricately involved in steroidogenesis, by catalyzing conversion of progesterone and 17-hydroxyprogesterone to 11-deoxycorticosterone and 11-deoxycortisol respectively which are substrates for mineralocorticoid and glucocorticoid production. A germline mutation in CYP21A2 causing complete absence of CYP21A2 activity leads to congenital adrenal hyperplasia (40). We describe a germline CYP21A2 variant c.1174G > A (p.A392T) in a patient who did not have typical features of classical CAH but clearly did exhibit reduced CYP21A2 transcript and 21-hydroxylase protein expression in both her corticotroph tumors. We acknowledge that we cannot say with certainty that the corticotroph tumor CYP21A2 mutation that we observed in this patient contributed to either formation of and/or growth of her tumors. However, we hypothesize that the reduced cortisol feed-back on her pituitary corticotroph cells may in some ways have predisposed her corticotroph cells to incur somatic mutations, such as those we observed in GPR162 and USP8 ultimately leading to tumorigenesis. In support of our hypothesis, rare germline CYP21A2 variants have been reported in patients with CD (41, 42), underpinning the role of dysregulated glucocorticoid feedback in proliferation and hypersecretion of corticotroph cells.

In summary, we report for the first time a double pituitary corticotroph tumor which harbored a G-protein coupled receptor (GP162) variant in one tumor and a novel ubiquitin specific protease (USP8) variant in the second corticotroph tumor in the setting of a background germline CYP21A2 variant. We propose a mechanism whereby chronic mild disruption in negative glucocorticoid feed-back on the pituitary corticotrophs facilitated secondary somatic variants and contributed to corticotroph tumor formation and/or growth.

## Figures and Tables

**Table 1:** Patient’s Pre and Post-Operative Biochemical Examine Results

	Patient’s Pre- operative Labs	Patient’s Post- Operative Lab	Reference Ranges
Prolactin	12.7 ng/mL	10.1 ng/mL	3–30 ng/mL
Midnight Salivary Cortisol	0.14 mcg/dL 0.39 mcg/dL 1.3 mcg/dL		10pm-1am: ≤0.09 mcg/dL
Cortisol	8am: 31.3 mcg/dL	6am: 1 9am: 2	Morning cortisol: 4–22 mcg/dL
Adrenocorticotropic Hormone (ACTH)	73 pg/mL	6am: 8 9am: 12	6–50 pg/mL
Thyroid Stimulating Hormone (TSH)	0.56 mIU/L		0.4–4.5 mIU/L
Free Thyroxine (FT4)	1.0 ng/dL	1.0 ng/dL	0.8–1.8 ng/dL
Insulin-like Growth Factor (IGF-1)	IGF-1: 214 ng/mL Z score: standard deviation (SD) 0.9	IGF-1: 118 ng/mL Z score: (SD) −0.4	IGF-1: 52–328 ng/mL Z score: SD −2.0 to 2.0
24-hour urine free cortisol (UFC)	24-hour UFC: 426.1 mcg 24-hour creatinine: 1.66g/24h, 1.93L urine volume		24-hour UFC: 4-50mcg/24hr 24-hour urine free creatinine: 0.50-2.15g/24h
1mg dexamethasone suppression test (DST)	8am cortisol: 28.8 mcg/dL		AM cortisol dexamethasone suppression test: <2 mcg/dL
17-OH progesterone		29 ng/dL	Follicular phase: 23–102 ng/dL
